# Tópicos Emergentes em Insuficiência Cardíaca: COVID-19 e Insuficiência Cardíaca

**DOI:** 10.36660/abc.20201081

**Published:** 2020-11-01

**Authors:** Livia Adams Goldraich, Odilson Marcos Silvestre, Edval Gomes, Bruno Biselli, Marcelo Westerlund Montera

**Affiliations:** 1 Hospital de Clínicas de Porto Alegre Porto AlegreRS Brasil Hospital de Clínicas de Porto Alegre, Porto Alegre, RS - Brasil; 2 Universidade Federal do Acre Rio BrancoAC Brasil Universidade Federal do Acre, Rio Branco, AC - Brasil; 3 Universidade Estadual de Feira de Santana Feira de SantanaBA Brasil Universidade Estadual de Feira de Santana, Feira de Santana, BA - Brasil; 4 Instituto do Coração da Universidade de São Paulo São PauloSP Brasil Instituto do Coração da Universidade de São Paulo, São Paulo, SP - Brasil; 5 Hospital Pró-Cardíaco Rio de JaneiroRJ Brasil Hospital Pró-Cardíaco, Rio de Janeiro, RJ - Brasil

**Keywords:** COVID-19, Insuficiência Cardíaca, Miocardite, Takotsubo, Lesão Miocárdica

## Introdução

Alguns autores têm proposto a denominação de “síndrome cardiovascular aguda pela COVID-19” para descrever as alterações do sistema cardiovascular associadas à infecção pelo SARS-CoV-2.^1^ Entre as diferentes manifestações, podem ocorrer injúria miocárdica, miocardite, infarto do miocárdio com coronárias normais (MINOCA), arritmias, Takotsubo, derrame pericárdico, insuficiência cardíaca (IC), além dos fenômenos tromboembólicos^2^^,^^3^ (Tabela 1). Enfatizaremos a injúria miocárdica, a miocardite, a síndrome Takotsubo e a ocorrência e peculiaridades da COVID-19 em portadores de IC.

**Tabela 1:** Espectro das manifestações cardiovasculares pela COVID-19

Fenótipo	Características importantes
Injúria miocárdica	Troponina elevada (> percentil 99)
Síndrome coronária aguda	Troponina elevada (> percentil 99) + sintomas + doença coronária obstrutiva
MINOCA	Troponina elevada (> percentil 99) + sintomas (sem doença coronária obstrutiva)
Miocardite ou miopericardite	Troponina elevada (> percentil 99) + sintomas + achados compatíveis na BEM ou RNM
Takotsubo	Troponina elevada (> percentil 99) + sintomas + ventriculografia típica
Arritmias	Taquiarritmias atriais, ventriculares ou bradiarritmias
Insuficiência cardíaca	IC crônica descompensada por COVID-19 ou IC aguda causada por um dos seguintes: SCA, MINOCA, miocardite ou Takotsubo
Derrame pericárdico	Muitas vezes associada à (mio)pericardite
Tromboembolismo	TVP, TEP, AVC ou embolia periférica

*MINOCA: infarto do miocárdio com coronárias normais; BEM: biópsia endomiocárdica; RNM: ressonância magnética; IC: insuficiência cardíaca; SCA: síndrome coronária aguda; TVP: trombose venosa profunda; TEP: tromboembolismo pulmonar; AVC: acidente vascular cerebral*.

### Injúria Miocárdica

O impacto da injúria miocárdica relacionada à infecção por SARS-CoV-2 foi reconhecido já no início da pandemia, com dados chineses e, subsequentemente, múltiplas coortes de diversos países, invariavelmente demonstrando aumento de mortalidade associado à elevação de troponina.^2^^,^^4^ Os potenciais mecanismos de envolvimento cardíaco em pacientes com COVID-19 são múltiplos e envolvem fatores diretos de infecção viral e, principalmente, indiretos de dano miocárdico. A presença do receptor da enzima de conversão da angiotensina 2 na superfície do cardiomiócito e das células endoteliais vasculares sugeria que o SARS-CoV-2 poderia levar ao dano tóxico pelo vírus e, consequentemente, miocardite.^5^ No entanto, um estudo alemão baseado em autópsias detectou cópias do vírus em células intersticiais e em macrófagos invadindo o miocárdio, mas não em cardiomiócitos.^6^ Além disso, a presença de genoma viral no coração não se associou a infiltrado inflamatório típico de miocardite, sugerindo que a infecção pelo SARS-CoV-2 não ocasiona um quadro celular inflamatório clássico. É possível que outras vias de injúria inflamatória possam desempenhar papel no dano miocárdico pelo vírus, envolvendo, particularmente, vasculite e ativação sistêmica da liberação de citocinas.

## Miocardite

Apesar desses dilemas em relação à fisiopatologia do dano miocárdico causado pela infecção por COVID-19, diversos casos de miocardite fulminante vêm sendo reportados.^5^^,^^7^ As apresentações clínicas parecem ser variáveis, porém semelhantes às manifestações de miocardites causadas por outros agentes virais, incluindo dor torácica, dispneia, arritmias, febre e algum grau de disfunção ventricular. O eletrocardiograma pode demonstrar alterações difusas do segmento ST, infra ou supradesnível de segmentos PR ou ST e, às vezes, pode mimetizar alterações compatíveis com infarto agudo com supradesnível de ST.^2^^,^^3^ As troponinas encontram-se elevadas, embora em valores absolutos menores do que os observados em síndromes coronarianas. A dosagem de peptídeos natriuréticos pode auxiliar na confirmação diagnóstica de miocardite, particularmente quando os níveis de troponina estiverem pouco alterados. Alterações difusas na motilidade ao ecocardiograma parecem ser mais comuns na suspeita de miocardite, comparativamente às síndromes isquêmicas agudas. O uso da ressonância magnética pode auxiliar na confirmação diagnóstica, apresentando padrão típico de comprometimento inflamatório. Parece haver uma alta proporção de pacientes com elevação de troponina e/ou alterações de eletrocardiograma que demonstram persistência de quadro inflamatório e/ou achados de miocardite subclínica na ressonância magnética cardíaca, mesmo após a recuperação da COVID-19 (aproximadamente 56 dias após quadro agudo).^8^

A concomitância de níveis elevados de troponinas, alterações no eletrocardiograma e presença de algum grau de disfunção ventricular configuram pior prognóstico na miocardite por SARS-CoV-2, embora qualquer evidência de injúria miocárdica deva ser considerada como marcador de risco para pacientes com diagnóstico de COVID-19, independentemente da suspeita de miocardite. Assim como no manejo da infecção por COVID-19 e suas múltiplas repercussões sistêmicas, a estratégia terapêutica específica para miocardite por SARS-CoV-2 baseia-se principalmente em tratamento de suporte sistêmico. O uso de imunomoduladores, como corticosteroides, e bloqueadores do receptor de interleucina 6, como tocilizumab, foi reportado em relatos de caso e em revisão sistemática recente e seus efeitos na COVID-19 ainda estão sob investigação.^9^^,^^10^ Arritmias merecem algum grau de monitorização, embora não haja tratamento específico recomendado no contexto da infecção por SARS-CoV-2.^11^

### Síndrome Takotsubo

Houve um aumento de cerca de cinco vezes na incidência de síndrome Takotsubo durante a pandemia por SARS-CoV-2, em comparação com as síndromes coronárias agudas, que tiveram redução dos casos no mesmo período. Uma coorte da Cleveland Clinic mostrou que, durante a pandemia, cerca de 8% dos casos que se apresentaram como síndrome coronária aguda eram Takotsubo, diferente da incidência de 1% relatada antes da pandemia.^12^ Os mecanismos fisiopatogênicos associados a esse aumento podem ser resultado direto do vírus, causando uma miocardite que mimetiza o Takotsubo (cardiomiopatia Takotsubo–*like*) ou, mais provável, o efeito do estresse psicológico imposto pela quarentena e pelo risco da pandemia, pela perda da interação social causada pelas regras de distanciamento e pelas consequências socioeconômicas da pandemia. As séries de caso mostram apresentação clínica semelhante àquelas relacionadas a outros fatores estressantes e mortalidade similar àquela descrita fora da situação de pandemia por COVID-19.

### COVID-19 em Pacientes com IC

A presença de IC no contexto da COVID-19 identifica um subgrupo de manejo complexo e de maior morbimortalidade. A IC pode representar tanto um fator de risco para uma pior evolução infecciosa quanto uma complicação cardiovascular grave causada pelo vírus SARS-CoV-2.^13^ A ativação da cascata inflamatória, a hiperestimulação do sistema neuro-humoral e a toxicidade viral direta representam alguns dos possíveis mecanismos fisiopatológicos para a IC aguda nova ou descompensada nesse cenário.

Pacientes hospitalizados com IC devem, preferencialmente, realizar pesquisa de SARS-CoV-2 em virtude da sobreposição de sinais e de sintomas e devem ser submetidos à avaliação minuciosa sobre o status volêmico, além de avaliação laboratorial, ecocardiográfica e radiológica. A COVID-19 pode ser entendida como uma síndrome inflamatória sistêmica e essa característica deve ser considerada na prescrição de vasodilatadores para indivíduos com IC aguda. A manutenção de medicações recomendadas pelas diretrizes deve ocorrer durante a internação naqueles com hemodinâmica preservada e normotensos. Outras estratégias, como a telemedicina, incluindo o telemonitoramento e as consultas virtuais, foram importantes no manejo da IC crônica e na prevenção infecciosa. Além de reduzir o risco de exposição viral, esses programas auxiliaram nas orientações preventivas para a COVID-19 e na identificação de pacientes sob risco de descompensação.^14^

### Considerações Finais

O espectro do acometimento cardíaco pela COVID-19 em pacientes com ou sem insuficiência cardíaca prévia é atualmente um conhecimento em evolução. Da mesma forma, as consequências a médio e longo prazo dos efeitos da infecção por SARS-CoV-2 no coração poderão trazer desdobramentos clínico-epidemiológicos relevantes, porém ainda pouco previsíveis. É provocativo considerar que possamos estar diante de uma nova etiologia de cardiomiopatia, que eventualmente poderá contribuir para o aumento da incidência de IC nos próximos anos.

### Lista de participantes do Heart Failure Summit Brazil 2020 / Departamento de Insuficiência Cardíaca - DEIC/SBC

Aguinaldo Freitas Junior, Andréia Biolo, Antonio Carlos Pereira Barretto, Antônio Lagoeiro Jorge, Bruno Biselli, Carlos Eduardo Montenegro, Denilson Campos de Albuquerque, Dirceu Rodrigues de Almeida, Edimar Alcides Bocchi, Edval Gomes dos Santos Júnior, Estêvão Lanna Figueiredo, Evandro Tinoco Mesquita, Fabiana G. Marcondes-Braga, Fábio Fernandes, Fabio Serra Silveira, Felix José Alvarez Ramires, Fernando Atik, Fernando Bacal, Flávio de Souza Brito, Germano Emilio Conceição Souza, Gustavo Calado de Aguiar Ribeiro, Humberto Villacorta Jr., Jefferson Luis Vieira, João David de Souza Neto, João Manoel Rossi Neto, José Albuquerque de Figueiredo Neto, Lídia Ana Zytynski Moura, Livia Adams Goldraich, Luís Beck-da-Silva, Luís Eduardo Paim Rohde, Luiz Claudio Danzmann, Manoel Fernandes Canesin, Marcelo Bittencourt, Marcelo Westerlund Montera, Marcely Gimenes Bonatto, Marcus Vinicius Simões, Maria da Consolação Vieira Moreira, Miguel Morita Fernandes da Silva, Monica Samuel Avila, Mucio Tavares de Oliveira Junior, Nadine Clausell, Odilson Marcos Silvestre, Otavio Rizzi Coelho Filho, Pedro Vellosa Schwartzmann, Reinaldo Bulgarelli Bestetti, Ricardo Mourilhe Rocha, Sabrina Bernadez Pereira, Salvador Rassi, Sandrigo Mangini, Silvia Marinho Martins, Silvia Moreira Ayub Ferreira, Victor Sarli Issa.
