# Supplementary material for: Tópicos Emergentes em Insuficiência Cardíaca: COVID-19 e Insuficiência Cardíaca
Source: Arq Bras Cardiol. 2020 Nov 1;115(5):942–4. [Article in Portuguese] doi: 10.36660/abc.20201081 (PMC8452204; doi:10.36660/abc.20201081)
Supplement: Translation

# Translation: Emerging Topics in Heart Failure: COVID-19 and Heart Failure

**Authors:** Livia Adams Goldraich, Odilson Marcos Silvestre, Edval Gomes, Bruno Biselli, Marcelo Westerlund Montera

**Affiliations:** 1 Porto AlegreRS Brazil Hospital de Clínicas de Porto Alegre, Porto Alegre, RS - Brazil; 2 Rio BrancoAC Brazil Universidade Federal do Acre, Rio Branco, AC - Brazil; 3 Feira de SantanaBA Brazil Universidade Estadual de Feira de Santana, Feira de Santana, BA - Brazil; 4 São PauloSP Brazil Instituto do Coração da Universidade de São Paulo, São Paulo, SP - Brazil; 5 Rio de JaneiroRJ Brazil Hospital Pró-Cardíaco, Rio de Janeiro, RJ - Brazil

**Keywords:** COVID-19, Heart Failure, Myocarditis, Takotsubo Cardiomyopathy, Myocardial Infarction

## Introduction

Acute COVID-19 cardiovascular syndrome has been the term proposed to describe changes in the cardiovascular system associated with SARS-CoV-2 infection.^1^ The most common manifestations of cardiovascular involvement are myocardial injury, myocarditis, myocardial infarction with nonobstructive coronary arteries (MINOCA), arrhythmias, Takotsubo syndrome, pericardial effusion, heart failure (HF), and thromboembolic phenomena (Table 1).^2^^,^^3^ Here, we emphasize myocardial injury, myocarditis, Takotsubo syndrome and the occurrence of COVID-19 in patients with preexisting HF.

**Table 1:** Spectrum of cardiovascular manifestations associated with COVID-19

Phenotype	Important characteristics
Myocardial injury	Elevated troponin (> 99th percentile)
Acute coronary syndrome	Elevated troponin (> 99th percentile) + symptoms + obstructive coronary artery disease
MINOCA	Elevated troponin (> 99th percentile) + symptoms + no obstructive coronary artery disease
Myocarditis or myopericarditis	Elevated troponin (> 99th percentile) + symptoms + EMB or CMR findings
Takotsubo syndrome	Elevated troponin (> 99th percentile) + symptoms + typical ventriculography
Arrhythmias	Atrial or ventricular tachyarrhythmias; bradyarrhythmias
Heart failure	Acutely decompensated chronic HF triggered by COVID-19 or de novo acute HF caused by one of the following: ACS, MINOCA, myocarditis, or Takotsubo syndrome
Pericardial effusion	Often associated with pericarditis or myopericarditis
Thromboembolism	DVT, PE, stroke, or peripheral emboli

*MINOCA: myocardial infarction with nonobstructive coronary arteries; EMB: endomyocardial biopsy; CMR: cardiac magnetic resonance; HF: heart failure; ACS: acute coronary syndrome; DVT: deep vein thrombosis; PE: pulmonary embolism*.

### Myocardial Injury

The impact of myocardial injury associated with SARS-CoV-2 infection was recognized early in the pandemic, when data from China and, subsequently, from multiple cohorts in different countries invariably showed an increase in mortality associated with elevated serum troponin levels.^2^^,^^4^ The mechanisms of cardiac involvement in patients with COVID-19 are multiple and include factors directly related to viral infection and, mainly, indirectly related to myocardial damage. The presence of the angiotensin-2 converting enzyme receptor on the surface of cardiomyocyte and vascular endothelial cells suggested that SARS-CoV-2 could cause toxic damage and, consequently, myocarditis.^5^ However, a German study based on autopsy cases detected copies of the virus in interstitial cells and macrophages invading the myocardium, but not in cardiomyocytes.^6^ In addition, the presence of viral genome in the heart was not associated with inflammatory infiltrates typical of myocarditis, suggesting that SARS-CoV-2 may not cause a classic cell-mediated inflammatory condition. It is possible that other inflammatory injury pathways may play a role in myocardial damage by the virus, involving, in particular, vasculitis and systemic activation of cytokine release.

### Myocarditis

Despite the uncertainty surrounding the pathophysiology of myocardial injury caused by SARS-CoV-2 infection, several cases of fulminant myocarditis have been reported.^5^^,^^7^ Clinical manifestations appear to be similar to those of myocarditis caused by other viruses and include chest pain, dyspnea, arrhythmia, fever, and ventricular dysfunction. The electrocardiogram (ECG) shows diffuse ST-segment abnormalities and depression or elevation of the PR and ST segments; at times, it can mimic pathologic changes compatible with ST-segment elevation myocardial infarction.^2^^,^^3^ Troponin is usually elevated, but at lower levels than those observed in acute coronary syndromes. Natriuretic peptides can contribute to the diagnostic confirmation of myocarditis, especially when troponin levels are only slightly increased. Diffuse changes in myocardial wall motion on echocardiography are more common in myocarditis than in acute ischemic syndromes, in which segmental changes are usually observed. Magnetic resonance imaging can be useful for diagnostic confirmation by revealing a typical pattern of inflammatory involvement. A high proportion of patients with elevated troponin and/or ECG changes appear to have persistent inflammatory abnormalities compatible with subclinical myocarditis on cardiac magnetic resonance, even after clinical recovery from COVID-19.^8^

The concomitant occurrence of elevated troponin, ECG changes, and left ventricular dysfunction is associated with a worse prognosis in SARS-CoV-2 myocarditis, although any evidence of myocardial injury should be considered a risk marker for patients with COVID-19, regardless of suspected myocarditis. Similar to the management of COVID-19 and its multiple systemic repercussions, specific therapeutic strategies for SARS-CoV-2 myocarditis are based mainly on systemic support. The use of immunomodulators, such as corticosteroids and interleukin-6 receptor antagonists (e.g. tocilizumab), has been described in case reports and in a recent systematic review.^9^^,^^10^ Their effects on COVID-19 are still under investigation. Although arrhythmias require some degree of monitoring, no specific antiarrhythmic treatment is currently recommended in the setting of SARS-CoV-2 infection.^11^

### Takotsubo Syndrome

The incidence of Takotsubo syndrome had a 5-fold increase during the COVID-19 pandemic, whereas acute coronary syndromes showed a decrease in the number of cases during the same period. A Cleveland Clinic cohort study showed that, during the pandemic, about 8% of the cases that presented as acute coronary syndrome were diagnosed as Takotsubo syndrome, different from the 1% incidence reported before the pandemic.^12^ Possible pathophysiologic mechanisms associated with this increase include a direct effect of the virus itself, causing myocarditis mimicking Takotsubo syndrome (Takotsubo-like cardiomyopathy), and, more likely, the effects of psychologic stress imposed by quarantine, risk of infection, reduced social interaction caused by social distancing, and socioeconomic consequences of the pandemic. Clinical presentation resembles that of other triggers, and mortality is similar to that reported in the prepandemic period.

### COVID-19 in Patients with HF

HF identifies a subgroup of patients with complex management issues and greater morbidity and mortality in the setting of COVID-19. HF represents both a risk factor for worse infectious outcomes and a serious cardiovascular complication of SARS-CoV-2 infection.^13^ Activation of the inflammatory cascade, hyperstimulation of the neurohumoral system, and direct viral toxicity are some of the possible pathophysiologic mechanisms for new-onset acute or decompensated HF in this scenario.

Patients hospitalized for HF should be tested for SARS-CoV-2 infection, due to overlapping signs and symptoms, and undergo a thorough assessment of volume status, in addition to laboratory, echocardiographic, and radiographic assessment. COVID-19 can manifest as a systemic inflammatory syndrome, and this feature should be considered when prescribing vasodilators for patients with acute HF. During hospitalization, the use of guideline-recommended medications should be maintained in patients with preserved hemodynamics and blood pressure. Other strategies, such as telemedicine, including telemonitoring and virtual consultations, have been important in the management of chronic HF and in infection prevention. In addition to reducing the risk of exposure to the virus, these strategies have helped to provide preventive counseling regarding COVID-19 and to identify patients at risk of decompensation.^14^

### Final Considerations

The spectrum of cardiac involvement in COVID-19, in patients with or without previous HF, is currently an evolving knowledge. Likewise, medium- and long-term consequences of the effects of SARS-CoV-2 infection on the heart may carry important clinical and epidemiological ramifications, but poorly predictable as yet. It is provocative to consider that we may be facing a potential new etiology of cardiomyopathy, which may contribute to an increase in the incidence of HF in the coming years.

### List of participants of the Heart Failure Summit Brazil 2020 / Heart Failure Department - Brazilian Society of Cardiology

Aguinaldo Freitas Junior, Andréia Biolo, Antonio Carlos Pereira Barretto, Antônio Lagoeiro Jorge, Bruno Biselli, Carlos Eduardo Montenegro, Denilson Campos de Albuquerque, Dirceu Rodrigues de Almeida, Edimar Alcides Bocchi, Edval Gomes dos Santos Júnior, Estêvão Lanna Figueiredo, Evandro Tinoco Mesquita, Fabiana G. Marcondes-Braga, Fábio Fernandes, Fabio Serra Silveira, Felix José Alvarez Ramires, Fernando Atik, Fernando Bacal, Flávio de Souza Brito, Germano Emilio Conceição Souza, Gustavo Calado de Aguiar Ribeiro, Humberto Villacorta Jr., Jefferson Luis Vieira, João David de Souza Neto, João Manoel Rossi Neto, José Albuquerque de Figueiredo Neto, Lídia Ana Zytynski Moura, Livia Adams Goldraich, Luís Beck-da-Silva Neto, Luís Eduardo Paim Rohde, Luiz Claudio Danzmann, Manoel Fernandes Canesin, Marcelo Bittencourt, Marcelo Westerlund Montera, Marcely Gimenes Bonatto, Marcus Vinicius Simões, Maria da Consolação Vieira Moreira, Miguel Morita Fernandes da Silva, Monica Samuel Avila, Mucio Tavares de Oliveira Junior, Nadine Clausell, Odilson Marcos Silvestre, Otavio Rizzi Coelho Filho, Pedro Vellosa Schwartzmann, Reinaldo Bulgarelli Bestetti, Ricardo Mourilhe Rocha, Sabrina Bernadez Pereira, Salvador Rassi, Sandrigo Mangini, Silvia Marinho Martins, Silvia Moreira Ayub Ferreira, Victor Sarli Issa.
